# Phenotypically sorted highly and weakly migratory triple negative breast cancer cells exhibit migratory and metastatic commensalism

**DOI:** 10.1186/s13058-023-01696-3

**Published:** 2023-08-30

**Authors:** Lauren A. Hapach, Wenjun Wang, Samantha C. Schwager, Devika Pokhriyal, Emily D. Fabiano, Cynthia A. Reinhart-King

**Affiliations:** 1 Nancy E. and Peter C. Meinig School of Biomedical Engineering, Cornell University, Ithaca, NY 14853 USA; 2 Department of Biomedical Engineering, Vanderbilt University, Nashville, TN 37212 USA

**Keywords:** Collective migration, Single cell migration, Leader–follower, Spheroid, Metastasis, Intratumor heterogeneity, Triple negative breast cancer

## Abstract

**Background:**

Intratumor heterogeneity is a well-established hallmark of cancer that impedes cancer research, diagnosis, and treatment. Previously, we phenotypically sorted human breast cancer cells based on migratory potential. When injected into mice, highly migratory cells were weakly metastatic and weakly migratory cells were highly metastatic. The purpose of this study was to determine whether these weakly and highly migratory cells interact with each other in vitro or in vivo*.*

**Methods:**

To assess the relationship between heterogeneity in cancer cell migration and metastatic fitness, MDA-MB-231 and SUM159PT triple negative breast cancer cells were phenotypically sorted into highly migratory and weakly migratory subpopulations and assayed separately and in a 1:1 mixture in vitro and in vivo for metastatic behaviors. Unpaired, two-tailed Student’s *t*-tests, Mann–Whitney tests, ordinary, one-way ANOVAs, and Kruskal–Wallis *H* tests were performed as appropriate with *p* < 0.05 as the cutoff for statistical significance.

**Results:**

When highly and weakly migratory cells are co-seeded in mixed spheroids, the weakly migratory cells migrated farther than weakly migratory only spheroids. In mixed spheroids, leader–follower behavior occurred with highly migratory cells leading the weakly migratory cells in migration strands. When cell suspensions of highly migratory, weakly migratory, or a 1:1 mixture of both subpopulations were injected orthotopically into mice, both the mixed cell suspensions and weakly migratory cells showed significant distal metastasis, but the highly migratory cells did not metastasize significantly to any location. Notably, significantly more distal metastasis was observed in mice injected with the 1:1 mixture compared to either subpopulation alone.

**Conclusions:**

This study suggests that weakly migratory cells interact with highly migratory cells in a commensal fashion resulting in increased migration and metastasis. Together, these findings indicate that cancer cell subpopulation migration ability does not correlate with metastatic potential and that cooperation between highly migratory and weakly migratory subpopulations can enhance overall metastatic fitness.

## Introduction

Intratumor heterogeneity can complicate cancer diagnosis and treatment and contribute to recurrence [1]. While the clinical impacts of intratumor heterogeneity are recognized, less is understood about how intratumor heterogeneity affects phenotypic behaviors such as migration and metastasis. While metastasis is a dynamic, multistep process, many studies have focused on the initial steps of local dissemination, where cancer cells adopt a motile phenotype to leave the primary tumor and migrate through the stroma [2]. Collective cell migration is the predominant migration mode observed in clinical samples and is associated with worsened patient prognosis in numerous cancer types; however, far less is known about this mode of migration compared to single cell migration [3–5]. In collective migration, the external chemical and physical cues and intracellular signaling and mechanotransduction events that dictate single cell migration are integrated across cohesive sheets, strands, or streams of coordinated migrating cells [6, 7]. While both single and collective cancer cell migration have been simultaneously observed in the same patients’ samples [8], it remains challenging to parse apart the relative contributions of each cell’s spatiotemporally unique interactions with the microenvironment and their intrinsic genetic disposition to determine whether this observed spectrum of migration modes reflects cellular plasticity or phenotypic diversity.

To address this problem, we previously sorted triple negative breast cancer (TNBC) cell lines into subpopulations based on migratory ability and found that surprisingly, in an orthotopic mouse model, weakly migratory cells metastasized significantly more than their highly migratory counterparts [9, 10]. These studies and numerous others [11–14] highlight the utility of phenotypic sorting methods in parsing apart intratumor heterogeneity to assess which cancer cells are ultimately critical for metastasis. Additionally, our group and others have studied leader–follower behavior, a pattern of collective migration where highly motile cells enable or guide directed migration of less motile cells [11, 14–18]. Importantly, this form of collective migration can facilitate the metastasis of other less motile subpopulations, introducing heterogeneity into metastatic sites and potentially imparting advantages such as enhanced survival and chemoresistance [10, 18–21]. As such, there is a need to better understand how individual cell phenotypes contribute to dissemination and metastasis.

In this study, we use phenotypically sorted highly migratory (MDA^+^, SUM^+^) and weakly migratory (MDA^−^, SUM^−^) human TNBC cell subpopulations. In an in vitro tumor spheroid model, highly migratory cancer cell spheroids migrated farther as both single cells and in migration strands compared to weakly migratory cancer cell spheroids, which migrated a shorter distance and in a predominantly collective fashion. When combined into 1:1 mixed spheroids, leader–follower behavior is observed with highly migratory cells preceding weakly migratory cells resulting in increased migration for weakly migratory cells compared to weakly migratory cancer cell-only spheroids. When injected in vivo orthotopically in a metastasis model, MDA^−^ cells metastasized more than MDA^+^ cells and MDA^−^ metastasis is increased when co-injected with MDA^+^ cells. These data suggest that highly migratory and weakly migratory cancer cell subpopulations cooperate in a commensal fashion to enhance overall metastatic fitness.

## Methods

### Cell culture and plasmids

MDA-MB-231 breast adenocarcinoma cells (HTB-26, ATCC, Rockville, MD) were maintained in DMEM with high glucose (25 mM; Life Technologies, Grand Island, NY) supplemented with 10% fetal bovine serum (FBS; Atlanta Biologicals, Flowery Branch, GA), 100 µg mL^−1^ streptomycin (Life Technologies), and 100 U mL^−1^ penicillin (Life Technologies). SUM159PT cells (BioIVT) were maintained in Ham’s F12 medium (Life Technologies) supplemented with 5% FBS, 10 mM HEPES, 1 µg/mL hydrocortisone, 5 µg/mL insulin, 100 µg mL^−1^ streptomycin, and 100 U mL^−1^ penicillin. All cell culture and time-lapse imaging were performed in a humidified environment at 37 °C and 5% CO_2_. FUW-GFP-E2A-fluc, FUW-mCherry-E2A-rluc, FUW-E-cadherin-E2A-mCherry plasmids were created in-house, and all subpopulations were stably transduced prior to in vitro and in vivo studies. Lentiviral particles were prepared and cells were transduced as described previously [22].

### Phenotypic cell sorting

To purify subpopulations based on migration ability, parental MDA-MB-231 cells (MDA^PAR^) or parental SUM159PT cells (SUM^PAR^) were seeded in a transwell migration assay as described previously [9]. Briefly, a coating of 1 mg mL^−1^ collagen gel (~ 10 μm thickness) was polymerized in a 6-well plate transwell insert with 8-μm pores (Corning) for 20 min. Cells were seeded in the collagen-coated transwell insert at 40,000 cells cm^−2^ in low-serum medium (0.5% FBS), and the insert was placed in a 6 well plate containing complete medium. On day 2, the low-serum medium was refreshed. On day 4, highly migratory (MDA^+^, SUM^+^) and weakly migratory (MDA^−^, SUM^−^) cells were collected separately with 0.25% Trypsin–EDTA from the bottom and top compartments, respectively. Twenty additional rounds of sorting were performed to further purify subpopulations. Subpopulations were used in experiments for up to 20 passages following purification without discernible changes in behavior.

### Spheroid preparation and embedding

Spheroids were generated as previously described [16]. Briefly, cells were harvested and resuspended in spheroid compaction medium containing 0.25% methylcellulose (H4100; Stem Cell Technologies, Cambridge, MA), 4.5% horse serum (Life Technologies), 18 ng mL^−1^ hEGF (Life Technologies), 0.45 µg mL^−1^ hydrocortisone (Sigma-Aldrich, St. Louis, MO), 9 µg mL^−1^ insulin (Sigma-Aldrich), 90 ng mL^−1^ cholera toxin (Sigma-Aldrich), 90 U mL^−1^ penicillin and 90 µg mL^−1^ streptomycin in DMEM/F12 (Life Technologies). The cell suspension was seeded into a 96-well round-bottom microplate with 5000 cells in each well, which was then centrifuged at 300×*g* for 5 min at room temperature.

After 3 days of compaction, spheroids were embedded in 1.5 or 4.5 mg mL^−1^ type I collagen gels. Collagen gels were prepared as previously described [15]. Briefly, type I collagen was acid-extracted from rat tail tendons (BioIVT, Westbury, NY), purified via centrifugation and lyophilization, and reconstituted at 10 mg mL^−1^ in 0.1% acetic acid. Stock collagen solution was diluted to either 1.5 or 4.5 mg mL^−1^ by gently mixing with ice-cold medium, and the solution was neutralized to pH 7.0 with 1 N NaOH. Spheroids were removed from culture plates and individually embedded within 500 µL collagen gels in glass-bottom 24-well plates (MatTek, Ashland, MA). After 45 min of gel polymerization at 37 °C, gels were overlaid with 500 µL of complete medium.

### Microscopy

Static or time-lapse imaging were carried out with a Zeiss LSM800 confocal microscope, equipped with an environment control chamber. A 10X dry lens N.A. = 0.3 was used to image embedded tumor spheroids with 20-µm-interval Z-stacks.

### Mice

Six–eight week old female NOD.Cg-*Prkdc*^scid^ *Il2rg*^tm1Wjl^/SzJ (NSG) immunodeficient mice (The Jackson Laboratory) were injected with 1 × 10^6^ MDA^+^, MDA^−^, or a 1:1 mix of MDA^+^: MDA^−^ cells subcutaneously at the mammary gland. For MDA^+^ and MDA^−^ only conditions, cells were tagged with a GFP plasmid. For the 1:1 mixed condition, half of the mice were injected with MDA^+^  + GFP and MDA^−^ + mCherry and the other half were injected with MDA^+^  + mCherry and MDA^−^ + GFP to control for any potential plasmid-dependent differences. At 4 weeks or when primary tumors approached 200 mm^3^ in volume, primary tumor removal surgery was performed following sterile surgical techniques. Four weeks after tumor removal, mice were euthanized and tissue samples were collected and fixed using 4% paraformaldehyde or snap frozen before processing for histological analysis.

### Data quantification and statistics

All statistical analysis for in vitro and in vivo studies was performed using GraphPad Prism Software. Unpaired, two-tailed Student’s *t*-tests, Mann–Whitney tests, ordinary, one-way ANOVAs, and Kruskal–Wallis H tests were performed as appropriate with *p* < 0.05 as the cutoff for statistical significance. All data are shown as mean ± SEM or box-and-whisker plots, where boxes represent medians and bars indicate 10th/90th percentiles with outliers represented as dots.

## Results

**Fig. 1:**
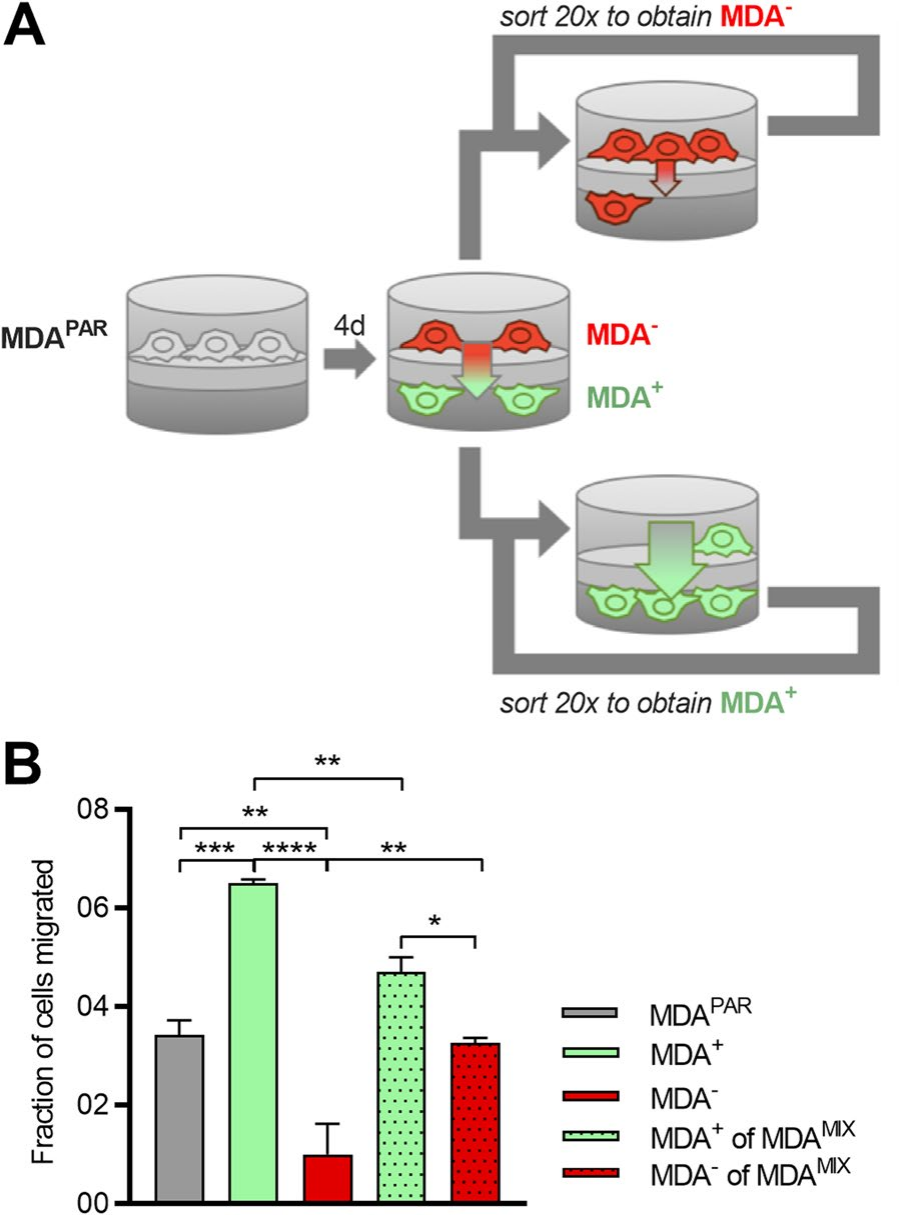
Phenotypic sorted cancer cells show differential invasion. **A** Schematic of transwell sorting assay that was performed to create MDA^+^ (green) and MDA^−^ (red) subpopulations. **B** Fraction of cells migrating through the transwell assay post-sorting. *p < 0.05, ** *p* < 0.01, ***p < 0.001, ****p < 0.0001

In prior work, we showed that phenotypic cell sorting of the human breast cancer cell line, MDA-MB-231 (MDA^PAR^), based on migration ability results in stable, distinct highly migratory (MDA^+^) and weakly migratory (MDA^−^) subpopulations [9]. Sorting was performed by seeding cells in low-serum media on a collagen-coated transwell insert (Fig. 1A). A serum gradient was established by filling the bottom reservoir of the transwell chamber with complete media. Over the course of 4 days, cells migrated through the transwell into the bottom chamber. After 4 days, cells on the top and bottom of the transwell were separately collected and seeded into fresh transwells. This process was repeated twenty times to purify highly migratory and weakly migratory subpopulations. Interestingly, when a 1:1 mixture of MDA^+^:MDA^−^ cells (MDA^MIX^) were co-seeded into transwells, migration of MDA^−^ cells in MDA^MIX^ transwells was increased compared to MDA^−^ only transwells while MDA^+^ cell migration in MDA^MIX^ transwells was unaffected compared to MDA^+^ only transwells (Fig. 1B). This data suggested that cooperative interactions where MDA^+^ cells enhance MDA^−^ cell migration may be occurring in MDA^MIX^ transwells.

**Fig. 2:**
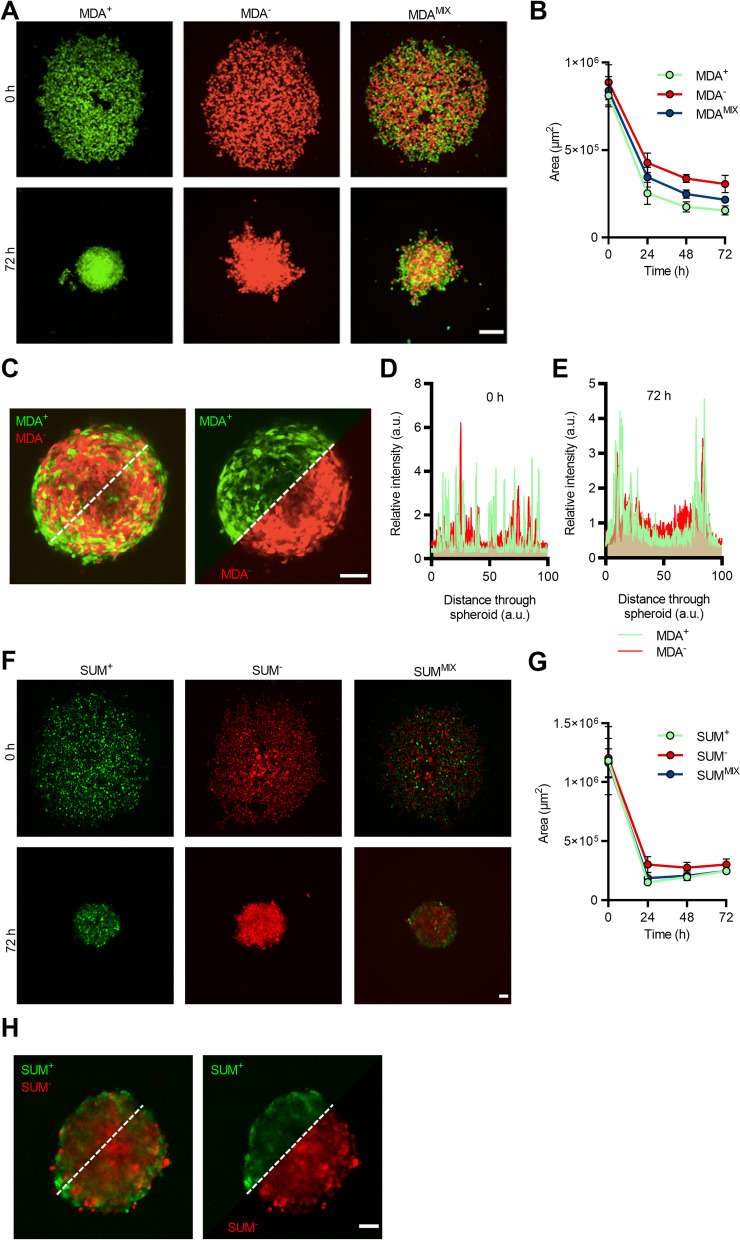
Phenotypic sorted cancer cells form in vitro tumor spheroids with differential compaction behavior. **A** Representative images of in vitro tumor spheroids immediately after seeding (0 h) and before embedding (72 h) with MDA^+^ (green), MDA^−^ (red), and MDA^MIX^ (1:1 MDA^+^:MDA^−^ co-culture) subpopulations; scale bar: 100 µm. **B** Compaction curve of MDA^+^, MDA^−^, and MDA^MIX^ spheroids from 0 to 72 h. **C** Representative image of fully compacted MDA^MIX^ spheroid (left) with individual MDA^+^ (green) and MDA^−^ (red) channels shown across spheroid diameter (right); scale bar: 50 µm. **D** Relative intensity histogram across diameter of MDA^MIX^ spheroid from (**A**) at 0 h. **E** Relative intensity histogram across diameter (white dotted line) of fully compacted MDA^MIX^ spheroid from (**C**) at 72 h. **F** Representative images of in vitro tumor spheroids immediately after seeding (0 h) and before embedding (72 h) with SUM^+^ (green), SUM^−^ (red), and SUM^MIX^ (1:1 SUM^+^:SUM^−^ co-culture) subpopulations; scale bar: 100 µm. **G** Compaction curve of SUM^+^, SUM^−^, and SUM^MIX^ spheroids from 0 to 72 h. **H** Representative image of fully compacted SUM^MIX^ spheroid (left) with individual SUM^+^ (green) and SUM^−^ (red) channels shown across spheroid diameter (right); scale bar: 100 µm

To further investigate the potential for cooperative interactions between the highly migratory and weakly migratory subpopulations, an in vitro tumor spheroid model was utilized. MDA^+^, MDA^−^, and MDA^MIX^ spheroids were formed over 3 days after seeding in a round-bottom 96-well plate (Fig. 2A). Spheroids compacted to different extents with MDA^+^ spheroids more compacted than MDA^MIX^ spheroids which were more compacted than MDA^−^ spheroids based on cross sectional area (Fig. 2B). At 72 h, MDA^+^ cells preferentially localized to the exterior of the spheroid while the MDA^−^ cells localized toward the spheroid core (Fig. 2C) in MDA^MIX^ spheroids. The distribution of MDA^+^ and MDA^−^ cells across the diameter of MDA^MIX^ spheroids were compared at 0 (Fig. 2D) and 72 h (Fig. 2E). At 0 h, both subpopulations were randomly distributed and then after compaction at 72 h, MDA^+^ cells have shifted toward the spheroid exterior while MDA^−^ cells have shifted toward the spheroid interior. To evaluate if these findings were specific to the MDA-MB-231 cell line, phenotypically sorted subpopulations from SUM159PT, another TNBC cell line, were tested. Highly migratory (SUM^+^), weakly migratory (SUM^−^), and a 1:1 mixture of SUM^+^:SUM^−^ (SUM^MIX^) spheroids were formed over 3 days (Fig. 2F). Like MDA-MB-231 subpopulations, spheroids compacted to different extents, with SUM^+^ and SUM^MIX^ compacting more than SUM^−^ (Fig. 2G). In SUM^MIX^ spheroids at 72 h, SUM^+^ cells also preferentially localized to the exterior of the spheroid with SUM^−^ localized toward the spheroid core (Fig. 2H). These results show that highly and weakly migratory cancer cell subpopulations, when formed into spheroids, compact with the more migratory cells at the spheroid periphery.

**Fig. 3:**
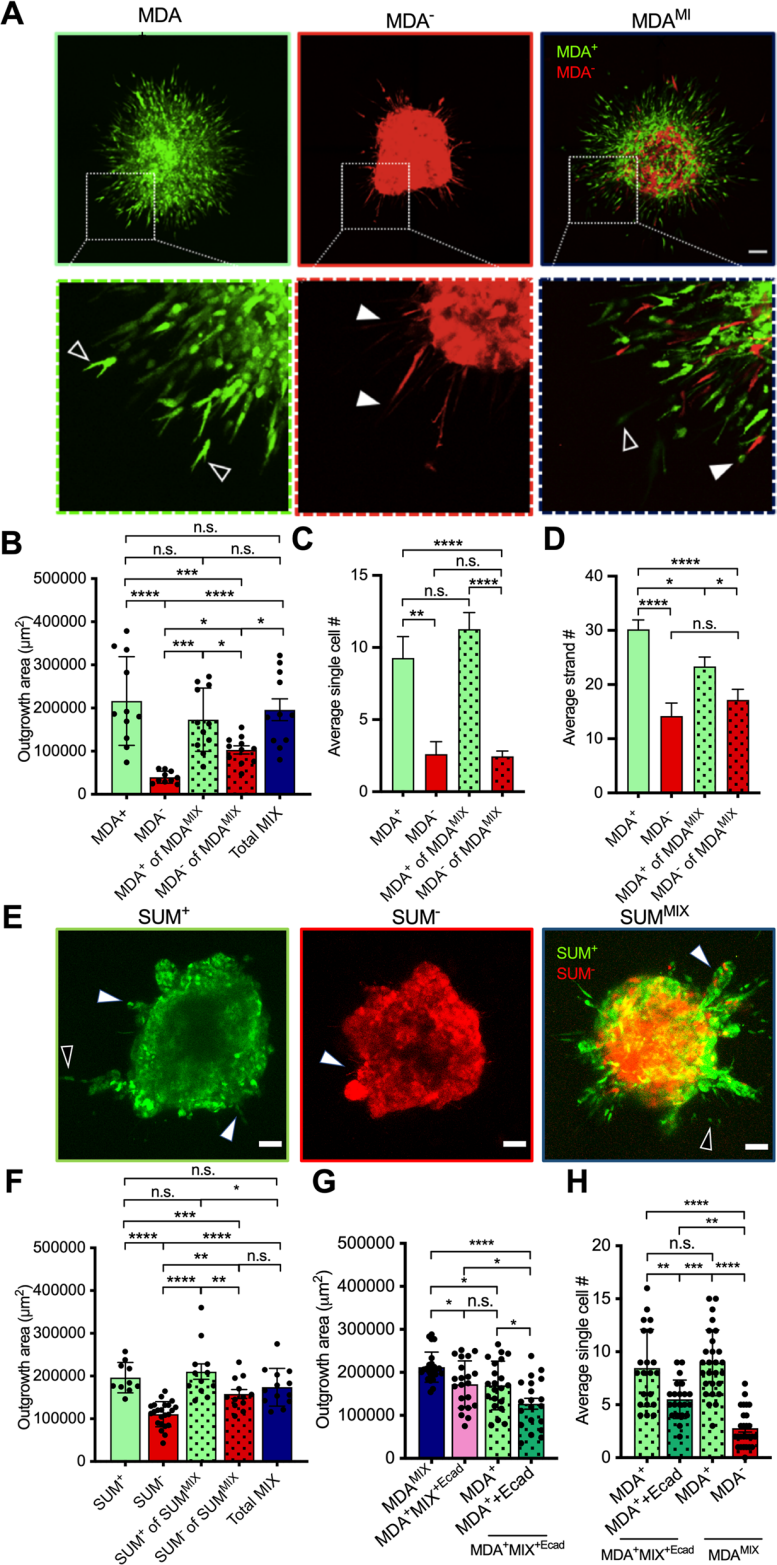
Phenotypic sorted cancer cells exhibit differential migration modes in tumor spheroid model. **A** Representative images of MDA^+^, MDA^−^, and MDA^MIX^ tumor spheroids in 1.5 mg mL^−1^ collagen at 24 h post-embedding. Black arrows inside inset images mark single cell migration while white arrows mark collective strand migration; scale bar: 100 µm. **B** Spheroid outgrowth at 24 h post-embedding. **C** Average number of single cells migrating from spheroids at 24 h post-embedding. **D** Average number of strands of migrating cells per spheroid at 24 h post-embedding. **E** Representative images of SUM^+^, SUM^−^, and SUM^MIX^ tumor spheroids in 1.5 mg mL^−1^ collagen at 72 h post-embedding. Black arrows mark single cell migration while white arrows mark collective strand migration; scale bar: 100 µm. **F** Spheroid outgrowth at 72 h post-embedding. **G** Outgrowth of spheroid generated with MDA^+^ and MDA^+^ cells with E-cadherin overexpression at 24 h post-embedding. **H** Average number of single cells migrating from spheroids at 24 h post-embedding. *p < 0.05, **p < 0.01, ***p < 0.001, ****p < 0.0001, n.s. = not significant

After characterizing tumor spheroid formation, we assessed cancer cell migration from the spheroids after embedding into 1.5 mg mL^−1^ 3D type I collagen matrix. After 24 h post-embedding, both MDA^+^ and MDA^MIX^ spheroids migrated out using both single and collective migration modes whereas MDA^−^ spheroids utilized predominantly collective migration (Fig. 3A). Further, both MDA^+^ and MDA^MIX^ spheroids migrated into the surrounding collagen matrix significantly farther than MDA^−^ spheroids (Fig. 3B). MDA^−^ cells in MDA^MIX^ spheroids migrated into the surrounding collagen significantly farther than MDA^−^ only spheroids (Fig. 3B). This suggests that the presence of MDA^+^ cells increased MDA^−^ cell migratory capability in MDA^MIX^ spheroids. MDA^+^ of MDA^MIX^ and MDA^+^ only spheroids exhibited no difference in spheroid outgrowth area (Fig. 3B) suggesting that the presence of MDA^−^ cells did not impact MDA^+^ migration ability in MDA^MIX^ spheroids.

When comparing single versus collective migration modes between spheroid conditions, single cell migration was more prevalent in MDA^+^ spheroids and MDA^+^ cells in MDA^MIX^ spheroids when compared to both MDA^−^ spheroids and MDA^−^ cells in MDA^MIX^ spheroids (Fig. 3C). Additionally, all spheroids used collective or strand-like or streaming migration behavior with no significant differences among spheroid conditions (Fig. 3D). We also observed different extents of spheroid migration and migration modes in the SUM159PT spheroids. After 72 h post-embedding, both SUM^+^ and SUM^MIX^ spheroids exhibited single and collective modes of migration whereas only low levels of collective migration were observed in SUM^−^ spheroids (Fig. 3E). Like MDA-MB-231 subpopulations, SUM^−^ of SUM^MIX^ exhibited greater outgrowth area than SUM^−^ alone while there was no significant difference in SUM^+^ cell migration when comparing SUM^MIX^ and SUM^+^ spheroids (Fig. 3F). Together, these results reveal that commensal interactions between highly and weakly migratory TNBC cells can promote the migration of weakly migratory cancer cells.

To gain a deeper understanding of the mechanism underlying the interaction between highly migratory cells and weakly migratory cells, we further investigated the role of E-cadherin, a cell–cell junction protein, in this interaction. Our previous study revealed that weakly migratory MDA cells express E-cadherin, while the highly migratory MDA subpopulation lacks E-cadherin expression [9]. Given the critical role of E-cadherin in the regulation of cell–cell interactions, we compared the outgrowth of MDA^MIX^ spheroids with that of spheroids generated by a 1:1 mixture of MDA^+^ cells and E-cadherin overexpressing MDA^+^ cells (referred to as MDA^+^E-cad). After 24 h of embedding, we observed that the spheroids generated by 1:1 mixture of MDA^+^ and MDA^+^ + E-cad cells (MDA^+^Mix^+Ecad^) exhibited significantly less outgrowth compared to MDA^MIX^ spheroids (Fig. 3G). Although there was no difference in the spheroid outgrowth area of MDA^+^ cells, MDA^+^ + E-cad cells migrated significantly less than MDA^+^ cells within the spheroids (Fig. 3G). This indicates that E-cadherin reduces the migration capability of MDA^+^ cells within the spheroids similar to that of MDA^−^ cells in the MDA^MIX^ spheroids. To investigate whether E-cadherin overexpression alters cell–cell interactions between MDA^+^ cells within the spheroids, we compared the average number of single cells observed 24 h after spheroid embedding. Single-cell migration was more prevalent in MDA^+^ cells than MDA^+^ + E-cad cells within spheroids (Fig. 3H). These findings suggest the involvement of E-cadherin in regulating the migration mode and interactions between highly migratory MDA cells and weakly migratory cells.

**Fig. 4:**
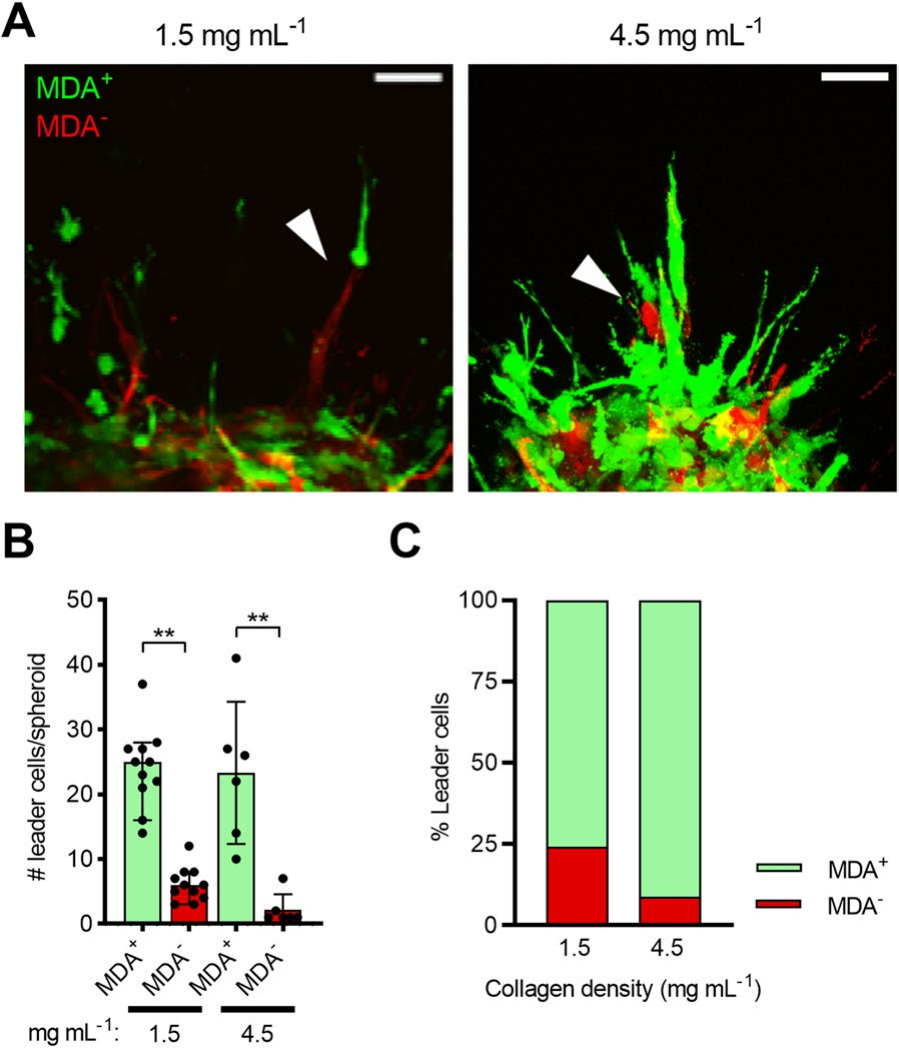
Co-culture spheroids exhibit leader–follower behavior. **A** Representative images of migration strands featuring leader–follower behavior in 1.5 and 4.5 mg mL^−1^ collagen gels 24 h post-embedding. **B** Average number of leader cells per spheroid. **C** Relative percentage of leader cells for MDA^+^ and MDA^−^ cells in MDA^MIX^ spheroids in 1.5 and 4.5 mg mL^−1^ collagen gels; **p < 0.01

These findings also led us to investigate the potential mechanism behind the enhanced migration distance of weakly migratory cells in mixed spheroids. When observing the relative location of cells within migration strands of MDA^MIX^ spheroids in 1.5 and 4.5 mg mL^−1^ collagen gels 24 h post-embedding, leader–follower behavior, where MDA^+^ cells lead and MDA^−^ cells follow, was observed in many of the migration strands (Fig. 4A). When quantified, there were significantly more MDA^+^ leader cells than MDA^−^ leader cells in both 1.5 and 4.5 mg mL^−1^ collagen matrices (Fig. 4B). Interestingly, when comparing the percentage of MDA^+^ leader cells in MDA^MIX^ spheroids at 1.5 and 4.5 mg mL^−1^, the percentage was significantly higher for the denser 4.5 mg mL^−1^ condition (Fig. 4C). Thus, these results suggest that leader–follower behavior where MDA^+^ leader cells facilitate MDA^−^ follower cell migration may contribute to the significantly farther distances that MDA^−^ cells in MDA^MIX^ spheroids can migrate compared to MDA^−^ cells in MDA^−^ only spheroids. We next considered whether these in vitro findings have in vivo implications for cancer metastatic potential.

**Fig. 5:**
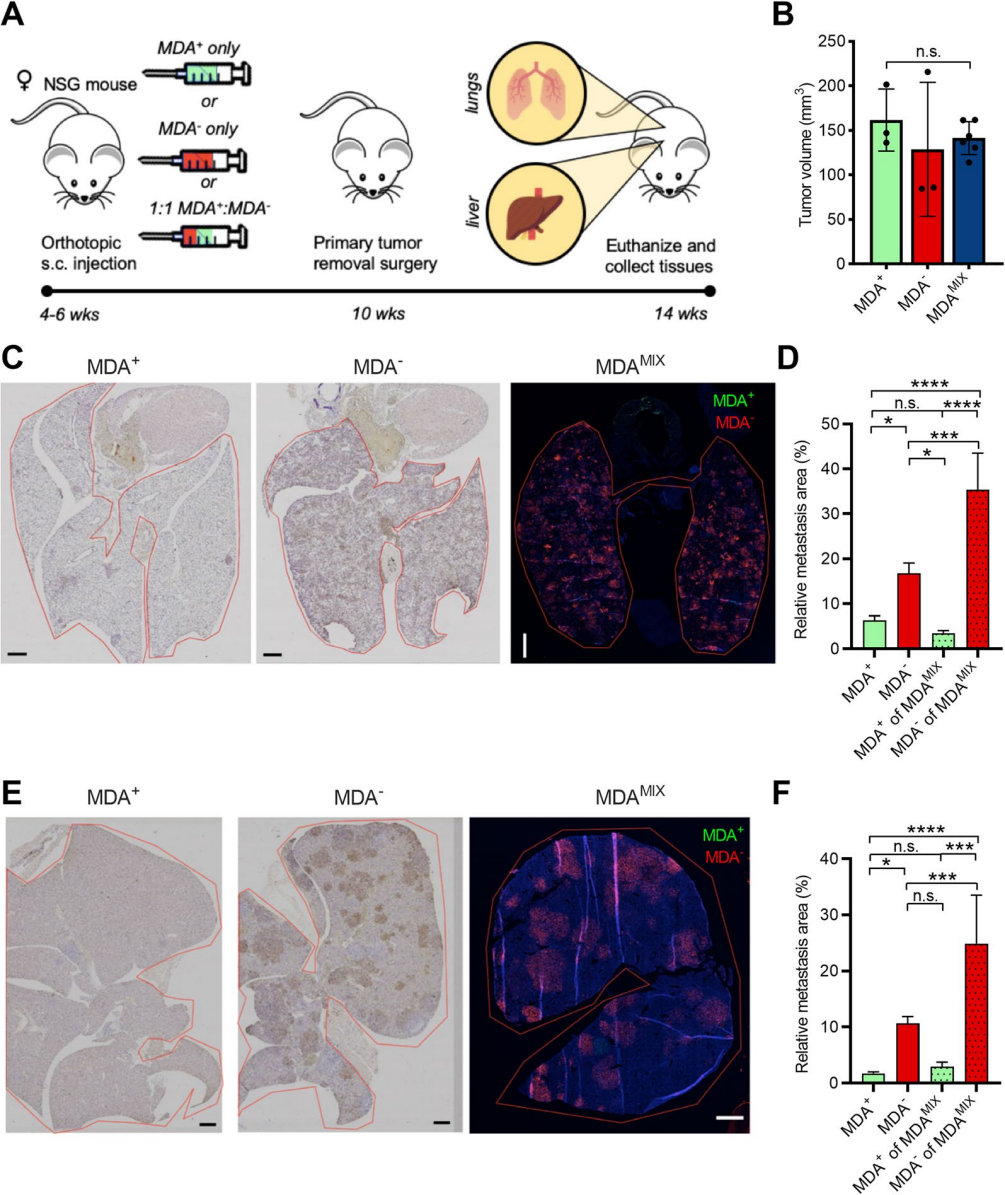
Phenotypic sorted cancer cells show commensal interactions leading to enhanced metastasis in vivo. **A** Schematic of orthotopic breast cancer spontaneous metastasis model where mice are injected with either MDA^+^, MDA^−^, or 1:1 MDA^+^:MDA^−^ (MDA^MIX^) cells, tumors are allowed to grow and then surgically removed, and metastasis is measured after collection of lung and liver tissues. **B** Average primary tumor volume at surgical removal (4 wks post-injection). **C** Representative GFP or mCherry staining of lungs from MDA^+^, MDA^−^, and MDA^MIX^-injected mice at study endpoint. **D** Average relative lung area covered by metastasis as evidenced by GFP and/or mCherry expression in MDA^+^ and MDA^−^ subpopulations. **E** Representative GFP or mCherry staining of livers from MDA^+^, MDA^−^, and MDA^MIX^-injected mice at study endpoint. **F** Average relative liver area covered by metastasis as evidenced by GFP and/or mCherry expression in MDA^+^ and MDA^−^ subpopulations. *p < 0.05, ** *p* < 0.01, ***p < 0.001, ****p < 0.0001, n.s. = not significant

To determine whether the commensal interactions between MDA^+^ and MDA^−^subpopulations observed in vitro affected metastatic potential in vivo, an orthotopic metastasis model was used where MDA^+^, MDA^−^, or MDA^MIX^ (1:1 mixture of MDA^+^: MDA^−^) cells were injected orthotopically at the mammary gland of 6–8 week old female NSG mice (Fig. 5A). At 4 weeks, primary tumors were surgically removed, and mice were monitored for an additional 4 weeks before sacrifice and tissue collection. Importantly, MDA^+^, MDA^−^, or MDA^MIX^ injections all resulted in tumors of similar volume (Fig. 5B). This suggests that the proliferation rates of MDA^+^, MDA^−^, and MDA^MIX^ cells were similar in vivo. GFP-positive and mCherry-positive cells were quantified in both lung and liver tissues and normalized by tissue area to calculate relative metastatic colonization. As previously observed [9], the percentage of metastatic colonization for MDA^−^-injected mouse tissues far exceeded those of MDA^+^ in both lungs (Fig. 5C–D) and liver (Fig. 5E–F). Interestingly, relative metastatic colonization for MDA^MIX^-injected mice tissues was significantly greater than those of MDA^−^-injected mice. This suggests that cooperative interactions occur between subpopulations in vivo resulting in enhanced overall metastatic potential. Importantly, in the MDA^MIX^ tissues, MDA^−^ cells were responsible for the vast majority of metastatic colonization while little to no metastasis was observed from MDA^+^ cells in MDA^MIX^ tissues. There was no significant difference in the percent area of metastatic colonization for MDA^+^ cells in lung or liver of MDA^+^- and MDA^MIX^-injected mice (Fig. 5D, F). In contrast, the relative metastatic colonization for MDA^−^ cells was significantly increased in both lung and liver of MDA^MIX^-injected mice compared to MDA^−^-injected mice (Fig. 5D, F). This data suggests that similar to the in vitro tumor spheroid model, the presence of highly migratory MDA^+^ cells in MDA^MIX^-injected mice enhanced the metastasis of weakly migratory MDA^−^ cells while not affecting MDA^+^ cell outcomes. Overall, these results suggest that commensal interactions between highly migratory and weakly migratory subpopulations occur in vivo and lead to increased metastatic fitness of MDA^−^ cells.

## Discussion

Using phenotypic sorting in both an in vitro and in vivo model of metastasis, we have described a commensal relationship between weakly and highly migratory phenotypically sorted cancer cell subpopulations, uncovering a relationship between cancer migration and metastasis in the context of intratumor heterogeneity. In prior work, we showed that weakly migratory cells metastasized in an E-cadherin dependent manner while highly migratory cells exhibited minimal metastasis until E-cadherin expression was induced [9]. Here, we show that when combined, the highly migratory cells aid in the migration of weakly migratory cells through leader–follower behavior. Additionally, overexpression of E-cadherin decreases the migration distance and relative amount of single cell migration of highly migratory cells. In vivo, the highly migratory cells enhance the already robust metastatic capability of the weakly migratory cells in vivo. Together, this work demonstrates that cells of varying migration ability may contribute to and promote the metastasis of other tumor cells.

**Fig. 6:**
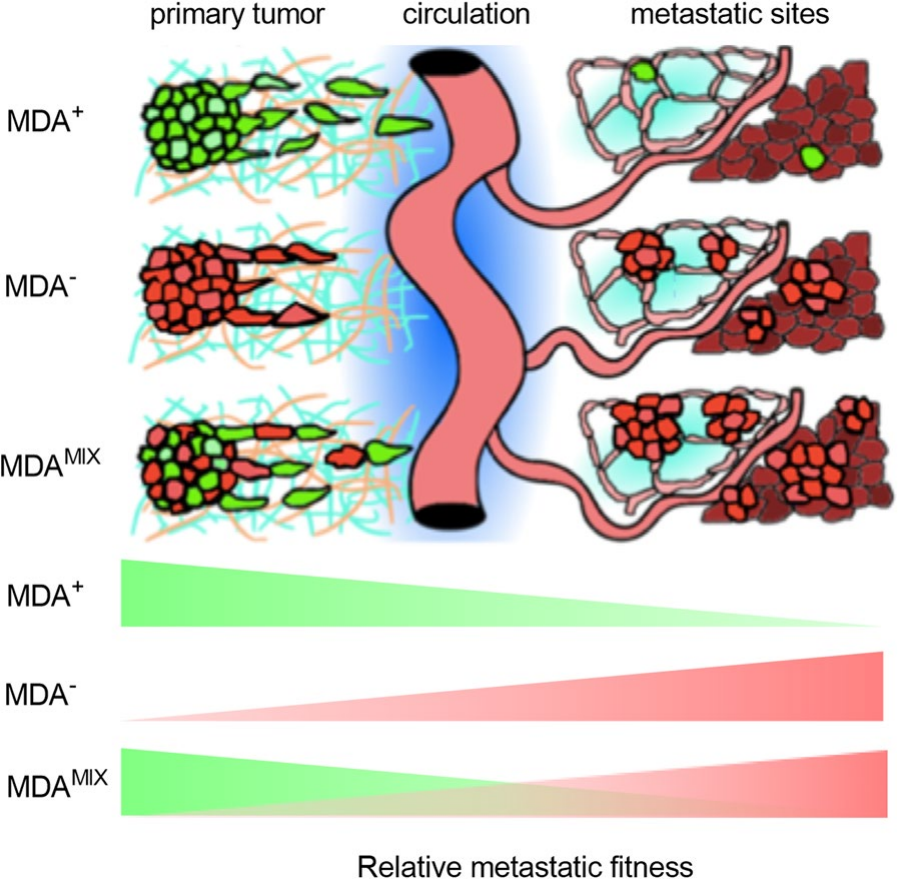
Commensal interaction between phenotypically sorted subpopulations hypothesized to be mediated by leader–follower behavior. **A** Schematic of hypothesized commensal interactions between MDA^+^ (green) and MDA^−^ (red) subpopulations in vivo where dissemination from the primary tumor by weakly migratory, highly metastatic MDA^−^ cells is enhanced by highly motile, weakly metastatic MDA^+^ cells in a leader–follower fashion resulting in more MDA^−^ cells arriving at metastatic sites and increased colonization compared to MDA^−^ cells alone. The proposed overall effect on metastatic fitness is depicted at the bottom of the schematic where MDA^+^ impart an early advantage during dissemination that is carried through to the final stage of metastasis, colonization, by MDA^−^ cells

The nature of cell–cell cooperation has varied from study to study, ranging from commensalism, where one population is benefitting from another, mutualism, where both populations receive a benefit from the other, and even synergism, where novel or enhanced characteristics emerge only when the two populations interact [23]. In our work, a commensal relationship between subpopulations was observed during migration from in vitro tumor spheroids embedded in 3D collagen matrix. Specifically, the migratory ability of weakly migratory cells was enhanced by highly migratory cells, and leader–follower behavior occurred in the mixed co-culture spheroids where highly migratory cells were leading weakly migratory cells (Fig. 6). While the cooperative benefit imparted by leader cells is generally enhanced migration of the follower cells, there are numerous cases of reciprocal benefits from follower cells toward leader cells, including enhanced proliferation, survival, and guidance cues [11, 24]. As a caveat, our study did not elucidate the specific cues responsible for this behavior in co-culture spheroids. Given that the percentage of MDA^+^ leader cells were significantly higher in 4.5 mg mL^−1^ collagen compared to 1.5 mg mL^−1^ collagen, highly migratory MDA^+^ cells may possess enhanced proteolytic or extracellular matrix (ECM) remodeling activity compared to weakly migratory MDA^−^ cells, allowing them to clear pathways in the denser collagen matrix thus enabling MDA^−^ follower cells to infiltrate further into the matrix without increased energetic burden. Interestingly, in our recent work examining the interactions between cancer-associated fibroblasts and MDA^+^ and MDA^−^ phenotypically sorted subpopulations, we found that MDA^+^ microvesicles were enriched for matrix metalloproteinase-14 (MMP-14) [10], a multi-functional proteinase that degrades ECM proteins such as collagen I and activates ERK signaling, both of which facilitate cancer cell invasion [25–28]. Conversely, MDA^−^ microvesicles were enriched for metalloprotease inhibitor-2 (TIMP-2) [10], which is associated with inhibition of matrix metalloproteases and decreased cancer cell invasion [29, 30]. As such, it is possible that MDA^+^ cells enhance migration of MDA^−^ cells by creating paths in the matrix.

To determine whether the interactions between strong and weak migrators affects metastasis, we orthotopically injected the cells and found that, consistent with our previous findings [9, 10], weakly migratory MDA^−^ cells metastasized significantly more to lungs and liver compared to highly migratory MDA^+^ cells. Interestingly, when injected as MDA^MIX^, there was a significant increase in metastasis to lung and liver for MDA^−^ cells in MDA^MIX^-injected mice compared to MDA^−^-injected mice. Based on our previous study, we know that MDA^−^ cells have the ability to form circulating tumor cell clusters in the circulation which are known to have enhanced metastatic potential compared to single cancer cells [9, 31]. In this study, it is possible that the MDA^+^-MDA^−^ leader–follower strands migrate from the primary tumor, intravasate into the circulation, and reach metastatic sites together as heterogeneous clusters. However, at the metastatic sites, we saw very minimal presence of MDA^+^ cells and no substantial heterogeneity in the metastatic lesions. This suggests that either the MDA^+^ cells do not accompany the MDA^−^ cells through the entirety of the metastatic cascade or that the MDA^+^ cells that reach a metastatic site alongside MDA^−^ cells do not have the capacity to establish or significantly contribute to a metastatic lesion or micrometastasis, even with the presence of metastasis-competent MDA^−^ cells. E-cadherin has been shown to play a key role in proliferation signaling at metastatic sites [32, 33]. As the weakly migratory MDA^−^ subpopulation has been previously shown to express E-cadherin while the highly migratory MDA^+^ subpopulation lacks E-cadherin expression [9], this could possibly explain the failure of MDA^+^ cells to significantly metastasize both alone and mixed with MDA^−^ cells. Together, these findings support the commensal interactions between weakly and highly migratory phenotypically sorted subpopulations.

When comparing these findings with other inter-cellular cooperation studies in cancer research, there are both striking similarities and differences that emphasize the difficulty in reaching universal conclusions around critical cell phenotypes for cancer metastasis. Many of these studies have characterized their cells of interest based on epithelial-to-mesenchymal-transition (EMT) characteristics [11, 14]. Previously, we found that weakly migratory cells possessed a more epithelial phenotype while their highly migratory counterparts had a more mesenchymal phenotype [9]. In agreement with our findings, many leader cells exhibit more mesenchymal characteristics compared to follower cells which are more epithelial in comparison [11, 14, 34]. Importantly, we did not observe polyclonal metastases containing both weakly and highly migratory cells in the MDA^MIX^-injected mice indicating that even in the presence of the highly metastatic MDA^−^ cells, MDA^+^ cells were unable to significantly colonize at the metastatic site. Coupled with our prior work indicating these cells are highly mesenchymal [9], we hypothesize MDA^+^ cells lack the plasticity to perform mesenchymal-to-epithelial-transition (MET) or reversion of EMT [35]. Recently, both experimentally and clinically, it has become more appreciated that EMT plasticity is advantageous for cancer metastasis [32, 36–39]. Our data suggest that the highly migratory subpopulation retains so few epithelial characteristics, they are unable to colonize distant sites, and while the weakly migratory subpopulation may appear relatively less aggressive based on migration ability, they are able to perform all stages of the metastatic cascade either alone or with assistance from cooperating cells.

Overall, our data support the possibility that a major advantage imparted by intratumor heterogeneity is the resulting phenotypic specialization of cancer cell subpopulations, and that by cooperating, subpopulations can optimize overall metastatic fitness [23]. An important caveat to our studies to date is that mesenchymal-like cells are known to possess enhanced chemoresistance and are often attributed with recurrence [20, 38, 40, 41]. So while we have demonstrated that weakly migratory cells are critical for successful metastasis, it is important to note that even the minimal metastasis that is achieved by the highly migratory subpopulation cannot be entirely discounted. With further studies toward (1) identification of critical cell phenotypes for cancer metastasis and (2) mechanistic understanding of cooperative events that facilitate or enhance these cells’ journey to colonize distant sites, more effective treatment strategies could be envisioned. While great strides have been made in cancer diagnosis and treatment, intratumor heterogeneity remains a daunting impediment to the ultimate goal of preventing or curing metastatic disease. Further understanding of how phenotypically diverse cancer cell subpopulations interact throughout disease progression is critical to overcoming this hurdle.

## Conclusions

Together, these results indicate that commensal interactions occur between these phenotypically sorted subpopulations to enhance overall cancer cell migratory and metastatic fitness. Future work should be performed to determine how prevalent leader–follower behavior is in heterogeneous parental cancer cell populations and by what chemical or physical means this behavior is mediated. More broadly, this project highlights the need for identification and characterization of subclones that are essential to metastasis as well as supportive and competing subclones and assessment of how these clonal interactions affect metastatic fitness at critical points of the metastatic cascade such as intravasation, survival in the circulation, and colonization.

## Data Availability

The datasets used and/or analyzed during the current study are available from the corresponding author on reasonable request.

## Abbreviations

ECM: Extracellular matrix
EMT: Epithelial-to-mesenchymal transition
FBS: Fetal bovine serum
HEPES: *N*-2-hydroxyethylpiperazine-*N*-2-ethane sulfonic acid
MDA^PAR^: Parental MDA-MB-231 cells
MDA^+^: Highly migratory MDA-MB-231 subpopulation cells
MDA^−^: Weakly migratory MDA-MB-231 subpopulation cells
MDA^MIX^: (1:1) Mix of weakly: highly migratory MDA-MB-231 subpopulation cells
MET: Mesenchymal-to-epithelial transition
NOD/SCID: Nonobese diabetic/severe combined immunodeficiency
SUM^PAR^: Parental SUM159PT cells
SUM^+^: Highly migratory SUM159PT subpopulation cells
SUM^−^: Weakly migratory SUM159PT subpopulation cells
SUM^MIX^: (1:1) Mix of weakly: highly migratory SUM159PT subpopulation cells
TNBC: Triple negative breast cancer
